# Effect of exercise interventions on cognitive function in breast cancer patients and survivors: a systematic review with meta-analysis

**DOI:** 10.1007/s12282-025-01757-9

**Published:** 2025-08-18

**Authors:** Chen-Sin Hung, Yi-Ting Cheng, Ruei-Hong Li, Charles H. Hillman, Neha P. Gothe, Feng-Tzu Chen, Hsing-Jung Yeh, Fei-Fei Ren, Yu-Kai Chang

**Affiliations:** 1 Department of Physical Education and Sport Sciences, National Taiwan Normal University, Taipei, Taiwan; 2 Department of Psychology, Northeastern University, Boston, MA USA; 3 Department of Physical Therapy, Movement, and Rehabilitation Sciences, Northeastern University, Boston, MA USA; 4 Institute for Cognitive and Brain Health, Northeastern University, Boston, MA USA; 5 Department of Kinesiology, National Tsing Hua University, Hsinchu, Taiwan; 6 Research Center for Education and Mind Sciences, National Tsing Hua University, Hsinchu, Taiwan; 7 Division of Gastroenterology and Hepatology, Department of Internal Medicine, Taipei Medical University Hospital, Taipei, Taiwan; 8 Division of Gastroenterology and Hepatology, Department of Internal Medicine, School of Medicine, College of Medicine, Taipei Medical University, Taipei, Taiwan; 9 Department of Physical Education, Beijing Language and Culture University, Beijing, China; 10 Social Emotional Education and Development Center, National Taiwan Normal University, Taipei, Taiwan

**Keywords:** Cancer, Cognitive function, Executive function, Exercise training, Cancer-related cognitive impairment

## Abstract

**Background:**

Exercise has been shown to facilitate cognitive function; however, data on changes in cognitive function in response to exercise interventions among breast cancer patients and survivors, who frequently experience cognitive impairment, have not been comprehensively synthesized. Therefore, this study aims to examine the impact of exercise interventions on cognitive function among breast cancer patients and survivors.

**Methods:**

Electronic databases including PubMed, Scopus, and Web of Science were searched from inception to December 25, 2024. A three-level meta-analysis was performed in R utilizing the standard mean difference. Moderators including cognitive function domains, sample characteristics (i.e., age and timing of exercise), and exercise regimen (i.e., frequency, intensity, type, session time, and length) were examined for subgroup analysis. The methodological quality and the certainty of evidence were evaluated using criteria of the Physiotherapy Evidence Database (PEDro) scale and the Grading of Recommendations Assessment, Development and Evaluation (GRADE) approach, respectively.

**Results:**

A total of 21 identified studies comprising 114 effect sizes were included in the final analysis. Overall, exercise demonstrated a positive small effect size on cognitive function in breast cancer patients and survivors (*g* = 0.22; 95% confidence interval [CI] 0.07–0.36; *p* < .001).

**Conclusions:**

Exercise demonstrated a facilitative effect on various cognitive functions among breast cancer patients during both treatment and survivorship. The non-significant moderation effects further suggest that diverse exercise regimens offer cognitive benefits. However, limited research highlights the need to identify optimal exercise modalities for cognitive enhancement in this population.

**Supplementary Information:**

The online version contains supplementary material available at 10.1007/s12282-025-01757-9.

## Introduction

Cancer, characterized by high mortality rates, is classified as a primary global cause of death by the World Health Organization (WHO) [1]. In 2020, about 19.3 million new cancer cases were reported, and nearly 18.1 million deaths [2]. Breast cancer, the most prevalent cancer among females [3], accounts for approximately 15.5% of all cancer-related deaths globally [4]. Despite advancements in medical technology enabling early detection and treatment, breast cancer survivors still experience diverse negative symptoms [5, 6].

Cancer-related cognitive impairment (CRCI) is a common concern among breast cancer patients and survivors [7], attributed to both treatment and the disease itself. Chemotherapy is recognized as a potential factor in CRCI, sometimes termed “chemobrain” [8]. Meta-analyses have consistently shown that breast cancer patients undergoing chemotherapy have a higher risk of experiencing cognitive impairment [7]. Patients exhibit poorer performance in language skills, visual-spatial abilities [9], and executive function [10]. These reduced cognitive functions may arise from chemotherapy-induced reductions in gray and white matter volumes [11].

Interestingly, cognitive impairment is not limited to the duration of chemotherapy alone [12]. While 75% of cases were observed during chemotherapy, approximately 40% occurred before chemotherapy, and nearly 60% were observed post-chemotherapy [12], suggesting a complex etiology beyond merely the influence of chemotherapy. CRCI is thought to be caused by numerous factors, including chemotherapy agents, inflammatory processes, genetic predisposition, and psychosocial impacts of diagnosis [7]. Importantly, patients at different treatment phases may face distinct physiological and psychological challenges, potentially leading to variations in both the mechanisms and manifestations of CRCI across treatment stages [7]. This multifactorial nature of CRCI highlights the importance of comprehensive approaches when investigating cognitive interventions for breast cancer survivors.

Exercise appears to serve as a non-pharmacological therapeutic approach for cognitive impairments in breast cancer patients [13]. Several studies have demonstrated the positive effects of exercise on cognitive function [14] and its role in reducing cognitive impairment [15]. The American College of Sports Medicine recommends that cancer patients avoid sedentary behavior [16]. Exercise may be more effective than other non-pharmacological interventions in attenuating cognitive impairment [17].

Notably, the benefits derived from exercise may vary depending on the exercise program performed (e.g., frequency, intensity, type, and session time). A meta-analysis focusing on executive function in older adults revealed that exercising 3 to 4 times weekly yields greater benefits than 1–2 times weekly [18]. High-intensity exercise appears to confer greater cognitive benefits to healthy adults compared to low-intensity exercise [19]. Studies associated with breast cancer have demonstrated that various types of exercise, including aerobic exercise [20], high-intensity interval exercise [21], and multi-component exercise [22], exhibit positive effects on cognitive function. However, whether exercise type moderates the effect remains unknown. Additionally, a study investigating the effects of acute exercise of varying session times on cognitive function in breast cancer patients found that the benefits differed based on the session time [23]. However, the effects of different session times of exercise intervention on cognitive function in breast cancer patients also remain unknown.

Breast cancer patients and survivors often endure cognitive impairment, and exercise emerges as a promising non-pharmacological intervention. However, the exercise effect on cognitive function may depend on the cognitive domains examined, sample characteristics, and the properties of the exercise intervention designed.

While previous studies have examined exercise effects on CRCI, some research gaps remain. Campbell, Zadravec [24] observed the benefits of physical exercise on cognitive function in cancer survivors; however, their study did not specifically target breast cancer patients and thus offers limited guidance for this population. Additionally, while Ren, Wang [25] focused on breast cancer survivors, their analysis emphasized specific exercise parameters (e.g., type). Therefore, this study aims to conduct a systematic review with meta-analysis to examine the impact of exercise interventions on cognitive function among breast cancer patients and survivors. Additionally, moderators regarding cognitive function domains, sample characteristics (i.e., age, and timing of exercise), and exercise regimen (i.e., frequency, intensity, type, session time, and length) were examined.

## Materials and methods

### Protocol and search strategy

This systematic review and meta-analysis was conducted in accordance with guidelines outlined in the Preferred Reporting Items for Systematic Reviews and Meta-Analyses [26]. The protocol was registered in PROSPERO (CRD42024551150). Electronic database searches were performed utilizing combinations of key terms, including ‘breast cancer’ ‘exercise’ and ‘cognitive function’ across PubMed, Scopus, and Web of Science (Supplement Table 1). The initial search encompassed studies published in peer-reviewed journals from inception until December 25, 2024.

### Eligibility criteria

The Participant, Intervention, Comparison, Outcome, and Study design (PICOS) principle was employed to establish the inclusion criteria. These criteria comprised: (1) female adults (≥ 18 years) diagnosed with breast cancer or survivors who had completed treatment; (2) exercise interventions, either supervised or unsupervised, excluding studies with additional non-exercise interventions; (3) control groups including no contact, waiting list, sham exercise, or active control; (4) outcomes measuring cognitive function, either self-reported or objectively assessed; (5) randomized controlled trials published in English. Single-arm and non-randomized studies, systematic reviews, case studies, and conference abstracts were excluded.

### Study selection and data extraction

Subsequent to the electronic database search and deduplication, the systematic screening protocol was implemented. Initially, abstract and title screening were conducted, followed by full-text review and data extraction. Each step was independently conducted by two authors (CSH and YTC). In case of disagreement, these two authors discussed the matter, and if consensus could not be reached, a third author (YKC) was consulted.

Extracted cognitive function feature included cognitive function measurement items and pre- and post-intervention cognitive function performance. Additionally, participant characteristics included age and timing of exercise, while exercise intervention characteristics included frequency, intensity, type, session time, and length. For detailed information, please refer to Supplemental Methods.

### Quality assessment

The evaluation of methodological quality was conducted independently by two researchers (CSH and YTC), classifying studies as excellent, good, fair, or poor quality. In instances of divergent outcomes, a third researcher (YKC) made the final determination. The Physiotherapy Evidence Database (PEDro) scale was employed for assessing the methodological quality, and the detailed criteria were presented in Supplement Table 2. A total score of 0–3 indicated poor quality, 4 or 5 suggested fair quality, 6–8 suggested good quality, while scores exceeding 9 indicated excellent quality [27].

The certainty of evidence was assessed using the Grading of Recommendations Assessment, Development and Evaluation (GRADE) approach, categorizing evidence quality as ‘high’, ‘moderate’, ‘low’, or ‘very low’, based on the following criteria: risk of bias, inconsistency, indirectness, imprecision, and other considerations. The detailed criteria were presented in Supplement Table 2. Additionally, after evaluating the quality of the evidence and considering other factors, recommendations for the strength and direction of the outcomes were proposed. These recommendations, classified as either ‘strong’ or ‘weak’, along with their direction (favor or against), were made regarding whether to adopt the intervention [28].

### Data synthesis and analysis

We conducted a multi-level meta-analysis using the “metafor” package (version 4.1.2) [29] in R [30]. This approach was selected to address the dependency of effect sizes, as multiple effect sizes were nested within individual studies. This multi-level meta-analysis framework accounts for three levels of variance: sampling variance (level 1), within-study variance (level 2), and between-study variance (level 3) [31].

Effect sizes were calculated using the standard mean difference (i.e., Hedges’ *g*) for between-groups pretest–posttest designs [32], with the assumption of a moderate correlation between pretest and post-test measures (*r* = 0.5) [33]. We assessed heterogeneity using the* I*^2^ statistic and Cochrane’s *Q*-test. Significance in heterogeneity was considered if the *p*-value of the *Q*-test was less than 0.1.* I*^2^ values were categorized as follows: ≤ 25% (low), 26–50% (mild), 51–75% (moderate), and > 75% (high) heterogeneity [34]. Publication bias was evaluated through visual inspection of a power-enhanced sunset funnel plot. A multilevel variant of Egger’s regression test was conducted to assess the presence of the small-study effect. Sensitivity analyses were conducted to identify and quantify the influence of outliers and influential meta-analytic reviews (i.e., studentized residuals and Cook’s distance) on the pooled effect estimates [35]. For more detailed information, please refer to Supplemental Methods.

Subgroup analyses were conducted to investigate moderators of the observed effects, including cognitive function based on cognitive domains: (a) self-report cognitive function—subjective assessments of cognitive performance measured via questionnaires, (b) basic cognitive function—attention, processing speed, and motor functioning [36], (c) high-order cognitive function—executive function [37] and learning [38], and (d) memory), age (i.e., middle-aged, older adults); timing of exercise (i.e., patients—individuals currently receiving medical treatment, survivors—individuals who have completed medical treatment, and mixed—both patients and survivors); exercise frequency (i.e., low, moderate, and high); intensity (i.e., low, moderate, vigorous, or not reported); type (i.e., aerobic exercise, resistance exercise, high-intensity interval training [HIIT], and multi-component exercise); session time (i.e., short, moderate, and long); and length (i.e., short and long). For more detailed information, please refer to the Supplemental Methods.

## Results

### Literature search

**Fig. 1:**
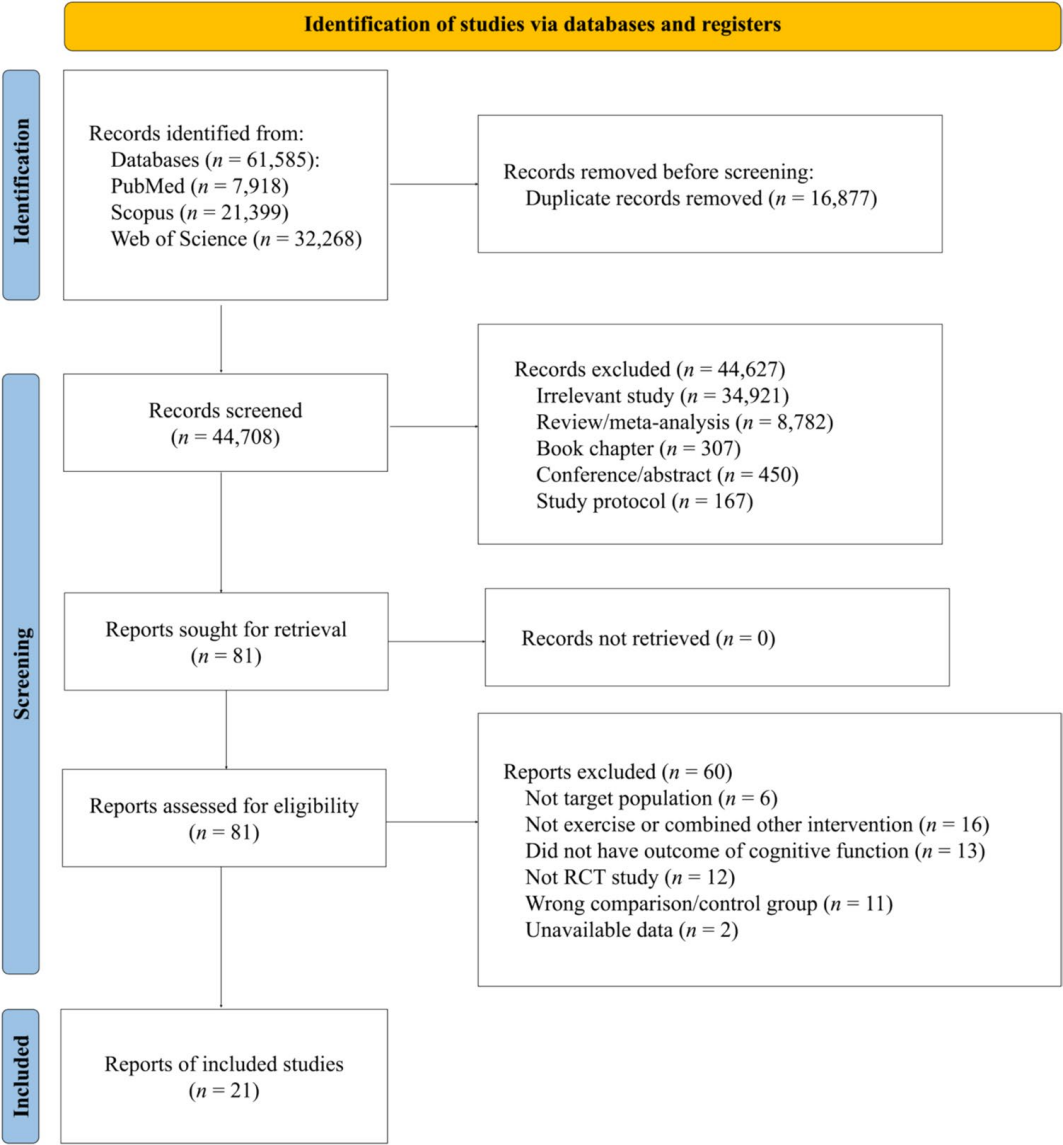
PRISMA flow chart of the study selection process

From the initial search, a total of 61,585 potentially relevant studies were identified. Following further review to exclude duplicates, irrelevant references, conference abstracts, as well as review and proposal articles, 81 full-text articles were retrieved. After evaluating these articles based on inclusion and exclusion criteria, the meta-analytic review included 23 randomized controlled trial studies. Among these, 2 studies were excluded due to unavailable data, resulting in a total of 21 studies [13, 14, 21, 22, 39–55]. An overview of the selection process is summarized in Fig. 1.

### Studies characteristics

The study characteristics are outlined in Table 1. There was considerable variation in sample sizes across randomized controlled trials, ranging from 17 to 240 participants. The overall sample size for the review was 1807 participants, including 967 and 840 in the exercise and control groups, respectively.

**Table 1:** Overview characteristics of available evidence from randomized controlled trials on exercise and cognitive function among breast cancer patients and survivors

Study	Sample characteristics (P)	Exercise characteristics (I)	Group (C)	Cognitive task (O)
Antunes, Joaquim [39]	Age: ≥ 18, EX: 49.66, UC: 51.02 Sex: F CS: I–III TS: Patients	Freq.: 3d/w Int.: 65–80% HRR Type: Multi-component Time: Reach 30 min Length: 20–24 weeks	Exercise (47) Usual care (46)	EORTC QLQ-C30
Bender, Sereika [14]	Age: < 80, EX: 61.44, CON: 62.75 Sex: F CS: I–IIIA TS: Mix	Freq.: 3d/w Int.: 60–80% HRR Type: Aerobic Time: 45–60 min Length: 24 weeks	Intervention (77) Control (76)	Grooved Pegboard Test Digit Vigilance Test Auditory Verbal Learning Test Rey Complex Figure Test Paired Associate Learning Test Rivermead Behavioral Memory Test Story Spatial Working Memory Rapid Visual Information Processing Delis Kaplan Color Word Interference Test Verbal Fluency Test One Touch Stockings of Cambridge Trail making Test B
Campbell, Kam [40]	Age: 40–65, EX: 53.2, CON: 51.4 Sex: F CS: I–IIIA TS: Survivors	Freq.: 4d/w Int.: 60–80% HRR Type: Aerobic Time: 30–45 min Length: 24 weeks	Exercise (10) Control (9)	FACT-Cog HVLT-R COWAT Animal Naming Stroop task TMT-A TMT-B
Chang, Yeh [41]	Age: > 20, QG: 51.91, CON: 52.77 Sex: F CS: I–II TS: Patients	Freq.: 5d/w Int.: NR Type: Multi-component Time: 35 min Length: 15 weeks	Qigong (33) Control (33)	EORTC QLQ-C30
Derry, Jaremka [42]	Age: 27–76, Yoga: 51.8, Wait: 51.3 Sex: F CS: 0–III TS: Survivors	Freq.: 2d/w Int.: NR Type: Multi-component Time: 1.5 h Length: 12 weeks	Yoga (100) Wait list (100)	BCPT Symptom Checklist
Galiano-Castillo, Cantarero-Villanueva [44]	Age: TEL: 47.4, CON: 49.2 Sex: F CS: I–IIIA TS: Survivors	Freq.: 3d/w Int.: NR Type: Multi-component Time: 1.5 h Length: 8 weeks	Telerehabilitation (40) Control (41)	EORTC QLQ-C30
Galiano-Castillo, Arroyo-Morales [43]	Age: TEL: 47.4, CON: 49.2 Sex: F CS: I–IIIA TS: Survivors	Freq.: 3d/w Int.: NR Type: Multi-component Time: 1.5 h Length: 8 weeks	Telerehabilitation (40) Control (41)	Auditory Consonant Trigrams TMT-A TMT-B TMT-B:A
Gokal, Munir [45]	Age: 27–74, INT: 52.08, CON: 52.36 Sex: F CS: I–III TS: Patients	Freq.: 5d/w Int.: Moderate intensity Type: Aerobic Time: 0.5 h Length: 12 weeks	Intervention (25) Control (25)	Cognitive Failures Questionnaire Digit span Sustained Attention to Response Task WAIS Block Design Stroop task
Hasnaoui, Van Hoye [54]	Age: > 18, EX: 58.8, CON: 57.7 Sex: F CS: I–IV TS: Mix	Freq.: 1 s/w Int.: NR Type: Multi-component Time: 1–1.5 h Length: 12 weeks	Intervention (11) Control (13)	EORTC QLQ-C30
Koevoets, Schagen [22]	Age: 30–75 INT: 52.1, CON: 52.5 Sex: F CS: I–III TS: Survivors	Freq.: 2 s/w Int.: Progressively increases Type: Multi-component Time: 2 h Length: 24 weeks	Intervention (91) Control (90)	RAVLT HVLT-R Corsi block-tapping test Digit Span Visual Reaction Time TMT-A TMT-B Tower of London Grooved pegboard MDASI-MM
Larkey, Roe [55]	Age: 40–75, QG: 57.7, SQG: 59.8 Sex: F CS: I–III TS: Survivors	Freq.: 1 s/w Int.: NR Type: Multi-component Time: 60 min Length: 12 weeks	QG (49) SQG (52)	FACT- Cog Letter Number Sequencing Digit Span
Mijwel, Backman [46]	Age: 18–70, RT + HIIT: 52.7, AE + HIIT: 54.4, CON: 52.6 Sex: F CS: I–IIIA TS: Patients	Freq.: 2d/w Int.: 70% 1 RM + 16–18 RPE (RT + HIIT)/13–15 RPE + 16–18 RPE (AE + HIIT) Type: Multi-component Time: 1 h Length:16 weeks	RT + HIIT (79) AE + HIIT (80) Control (81)	EORTC QLQ-C30 Piper Fatigue Scale
Moulton, Grazioli [47]	Age: > 18, EX: 50.55, CON: 45.15 Sex: F CS: I–III TS: Patients	Freq.: 2 s/w Int.: Reach 70–80% HRmax Type: Multi-component Time: 1 h Length: 16 weeks	Exercise (10) Control (10)	EORTC QLQ-C30 FA-12
Myers, Mitchell [48]	Age: > 18, QG: 52.89, GEN: 53.05, Sup: 56.18 Sex: F CS: I–III TS: Patients	Freq.: 14 s/w Int.: NR Type: Multi-component Time: 15 min Length: 8 weeks	Qigong (19) Gentle (20) Support (11)	FACT-Cog PROMIS RAVLT TMT-A TMT-B F-A-S
Northey, Pumpa [21]	Age: 50–75, HIIT: 60.3, MOD: 67.8, CON: 61.5 Sex: F CS: I–III TS: Survivors	Freq.: 3 s/w Int.: Reach 90% HR Max (HIIT)/9–13 RPE (Aerobic) Type: HIIT/Aerobic Time: 20–30 min Length: 12 weeks	HIIT (6) MOD (5) CON (6)	International Shopping List Groton Maze Learning Task N-back
Pasyar, Barshan Tashnizi [49]	Age: INT: 51.6, CON: 51.8 Sex: F CS: 0–III TS: Survivors	Freq.: 3 s/w Int.: NR Type: Multi-component Time: 45 min Length: 8 weeks	Intervention (20) Control (20)	EORTC QLQ-C30
Schmidt, Wiskemann [50]	Age: > 18, EX: 52.2, CON: 53.3 Sex: NR CS: I–IV TS: Patients	Freq.: 2d/w Int.: 60–80% 1-RM Type: Resistance Time: 1 h Length: 12 weeks	Exercise (52) Control (49)	EORTC QLQ-C30 Fatigue Assessment Questionnaire TMT
Schmidt, Weisser [51]	Age: 18–70, RT: 53, ET: 56, SC: 54 Sex: F CS: NR TS: Patients	Freq.: 2d/w Int.: 50% Max weight (RT)/ 11–14 RPE (ET) Type: Resistance/Aerobic Time: 1 h Length: 12 weeks	Resistance (21) Endurance (20) Standard Care (26)	EORTC QLQ-C30 D2 test
Steindorf, Schmidt [52]	Age: > 18, EX: 55.2, CON: 56.4 Sex: NR CS: 0–III TS: Patients	Freq.: 2d/w Int.: 60–80% 1-RM Type: Resistance Time: 1 h Length: 12 weeks	Exercise (80) Control (80)	EORTC QLQ-C30 Fatigue Assessment Questionnaire TMT
Vadiraja, Rao [53]	Age: 30–70, Yoga: 46.7, CON: 48.5 Sex: F CS: I–III TS: Patients	Freq.: 3 s/w Int.: NR Type: Multi-component Time: 1 h Length: 6 weeks	Yoga (44) Control (44)	EORTC QLQ-C30
Wei, Yuan [13]	Age: INT: 52, CON: 55 (Median) Sex: F CS: I–III TS: Patients	Freq.: 5 s/w Int.: NR Type: Multi-component Time: 0.5 h Length: 12 weeks	Intervention (35) Control (35)	FACT-Cog

Note: *EX* exercise group, *UC* usual care group, *CON* control group, *INT* intervention group, *MOD* moderate intensity continuous training group, *RT* resistance training group, *ET* endurance training group, *SC* standard care group, *SQG* sham qigong group, *QG* qigong group, *GEN* gentle exercise group, *TEL* telerehabilitation group, *NR* not reported, *Freq*. frequency, *Int*. intensity, *CS* cancer stage, *F* female, *TS* treatment state, *HIIT* high intensity interval training, *h* hour, *min* minute, *s* session, *w* week, *FACT-cog* Functional Assessment of Cancer Therapy—Cognitive Function, *HVLT-R* Hopkins Verbal Learning Test-Revised, *COWAT* Controlled Oral Word Association Test, *TMT* trail making test, *BCPT* Breast Cancer Prevention Trial, *WAIS* Wechsler Adult Intelligence Scale, *MDASI-MM* MD Anderson Symptom Inventory for multiple myeloma, *PROMIS* Patient-Reported Outcomes Measurement Information System, *RAVLT* Rey Auditory Verbal Learning Test

Regarding cognitive domains, the largest number of effect sizes were observed for self-report cognitive function (*n* = 38), followed by high-order cognitive function (*n* = 35), memory (*n* = 30), and basic cognitive function (*n* = 11). Regarding participant characteristics, the majority of effect sizes (*n* = 100) were derived from participants aged younger than 60 years and most participants were survivors (*n* = 77). Concerning exercise prescription variables, the largest number of effect sizes was from exercise interventions conducted at vigorous intensity (*n* = 44), utilizing multi-component exercise (*n* = 70), with each session lasting a short duration (≤ 30 min) (*n* = 45), and lasting for a short length (1–3 months) (*n* = 71).

### Result of quality assessment

The results of the methodological quality were presented in Table 2. The findings indicated that the majority of studies were of high quality (*n* = 19), with only one study rated as fair quality and another as poor quality.

**Table 2:** PEDro-scores for the studies included in the systematic review and meta-analysis

Study	1.Eligibility criteria*	2.Randomly allocated	3.Allocation concealed	4.Baseline similar	5.Blinding participants	6.Blinding therapists	7.Blinding assessors	8.85% Key outcome	9.Intention to treat	10.Between group	11.Point & variability measure	Total
Antunes, Joaquim [39]	1	1	1	1	0	0	0	1	1	1	1	7
Bender, Sereika [14]	1	1	0	1	0	0	1	1	1	1	1	7
Campbell, Kam [40]	1	1	1	1	0	0	1	1	0	1	0	6
Chang, Yeh [41]	1	1	1	1	0	0	0	1	0	1	1	6
Derry, Jaremka [42]	1	1	1	1	0	0	1	1	0	1	0	6
Galiano-Castillo, Cantarero-Villanueva [44]	1	1	1	1	0	0	1	1	1	1	1	8
Galiano-Castillo, Arroyo-Morales [43]	1	1	1	1	0	0	1	1	1	1	1	8
Gokal, Munir [45]	1	1	1	1	0	0	0	1	1	1	1	7
Hasnaoui, Van Hoye [54]	1	1	1	1	0	0	0	1	1	1	1	7
Koevoets, Schagen [22]	1	1	1	0	0	0	0	1	1	1	1	6
Larkey, Roe [55]	1	1	1	1	1	0	1	1	0	1	1	8
Mijwel, Backman [46]	1	1	1	1	0	0	0	1	1	1	1	7
Moulton, Grazioli [47]	1	1	1	1	0	0	1	1	1	1	1	8
Myers, Mitchell [48]	1	1	1	0	0	0	0	0	0	1	0	3
Northey, Pumpa [21]	1	1	1	1	0	0	0	1	0	1	1	6
Pasyar, Barshan Tashnizi [49]	1	1	1	1	0	0	1	0	0	1	1	6
Schmidt, Wiskemann [50]	1	1	1	1	0	0	0	0	1	1	1	6
Schmidt, Weisser [51]	1	1	1	1	0	0	0	0	0	1	1	5
Steindorf, Schmidt [52]	1	1	1	1	0	0	0	1	1	1	1	7
Vadiraja, Rao [53]	1	1	1	1	0	0	0	1	1	1	1	7
Wei, Yuan [13]	1	1	1	1	0	0	1	1	1	1	1	8

Note: *Not used to generate the total score, A total score out of 10 is determined from the number of criteria that are satisfied

According to the GRADE assessment, the overall certainty of evidence was rated as low quality, with a weak recommendation in favor. In this assessment, the risk of bias and inconsistency were assessed as serious, while indirectness, imprecision, and other considerations were assessed as not serious. Detailed information regarding other moderator assessments was presented in Supplement Table 3.

### Main analysis

**Fig. 2:**
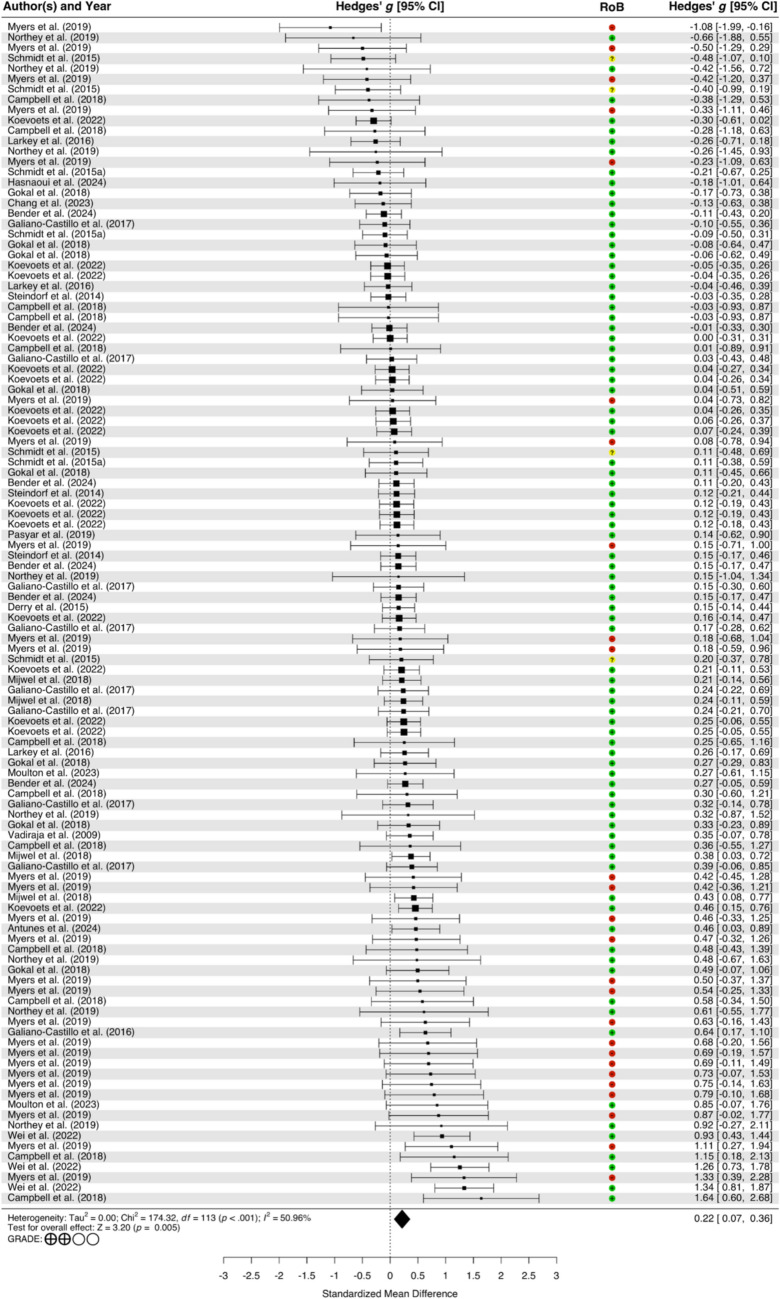
Displays 114 effect sizes from 21 included studies investigating the effects of exercise on cognitive function. Note: Effect sizes are presented in ascending order of magnitude and clustered by study in our three-level meta-analytic model. Multiple effect sizes from the same study represent different cognitive outcomes or exercise interventions, reflecting the hierarchical data structure (effect sizes nested within studies) in our analysis. *RoB* risk of bias, which was derived from PEDro scores, where high, moderate, and low methodological quality scores were converted to low, moderate, and high risk of bias, respectively, *GRADE* Grading of Recommendations Assessment, Development and Evaluation; ⨁⨁◯◯ = low certainty of evidence

The meta-analysis, from 21 studies with 114 effect sizes, revealed a significant positive effect of exercise on cognitive function (*g* = 0.22, 95% CI [0.07, 0.36]). Variance analysis indicated non-significant variability in level 2 effects (LRT = 0.00, *p* = 1.000, *I*^2^ = 0.00%), while level 3 effects showed significant variability (LRT = 22.33, *p* < 0.001, *I*^2^ = 50.96%). Heterogeneity across the studies was mild but not significant (*Q*_113_ = 174.32, *p* < 0.001, *I*^2^ = 50.96%). The results of the main analysis are outlined in Table 3, and the forest plot is presented in Fig. 2.

**Table 3:** Summary of the effect of exercise on cognitive function (CF)

Overall	*Q*	*n* ES	Hedges’ *g *(95% CI)	SE	*I*^2^
*Q*_113_ = 174.32,	*p* < .001	114	0.22 (0.07–0.36)	0.067	50.96%
*CF domains*		Subgroup analysis: *F*_3, 0.99_ = 1.34*, p* = .549			
Self-report cognitive function		38	0.29 (0.10–0.48)*	0.089	67.76%
Basic cognitive function		11	0.23 (0.06–0.41)*	0.068	0.00%
High-order cognitive function		35	0.12 (0.00–0.23)*	0.050	0.00%
Memory		30	0.09 (− 0.16 to 0.34)	0.101	22.24%
*Sample characteristics*					
*Age*		Subgroup analysis: *F*_1, 1.15_ = 2.00, *p* = .369			
Middle-aged (≤ 60 yrs)		100	0.23 (0.07–0.39)*	0.075	54.46%
Older adults (> 60 yrs)		14	0.11 (− 0.33 to 0.56)	0.028	0.00%
*Timing of exercise*		Subgroup analysis: *F*_2, 2.04_ = 0.70, *p* = .585			
Patients (during treatment)		30	0.25 (− 0.05 to 0.56)	0.134	70.99%
Survivors (after treatment)		77	0.21 (0.07–0.35)*	0.059	12.34%
Mix (patients and survivors)		7	0.03 (− 1.92 to 1.98)	0.121	0.00%
*Exercise regimens*					
*Frequency (week)*		Subgroup analysis: *F*_2, 7.60_ = 2.25, *p* = .171			
Low (1–2 times)		38	0.08 (−0.06 to 0.23)	0.060	21.47%
Moderate (3–4 times)		38	0.27 (0.11–0.43)*	0.066	8.47%
High (> 4 times)		38	0.40 (− 0.50 to 1.30)	0.264	69.12%
*Intensity*		Subgroup analysis: *F*_3, 2.39 _= 1.59, *p* = .385			
Light		7	− 0.07 (−1.40 to 1.27)	0.083	4.68%
Moderate		20	0.10 (− 0.24 to 0.43)	0.095	0.00%
Vigorous		44	0.20 (0.02–0.38)*	0.072	20.79%
Not reported		43	0.35 (0.01–0.70)*	0.146	56.43%
*Type*		Subgroup analysis: *F*_3, 2.14_ = 15.70*, p* = .053			
Aerobic exercise		32	0.09 (− 0.12 to 0.29)	0.070	0.00%
Resistance exercise		8	0.00 (− 0.22 to 0.21)	0.049	0.00%
HIIT		4	0.46 (0.06–0.85)*	0.051	0.00%
Multi-component exercise		70	0.30 (0.09–0.51)*	0.096	61.44%
*Session time*		Subgroup analysis: *F*_2, 6.94_ = 1.03,* p* = .407			
Short (≤ 30 min)		45	0.43 (− 0.38 to 1.25)	0.244	60.57%
Moderate (31–60 min)		28	0.12 (− 0.02 to 0.26)	0.061	22.44%
Long (> 60 min)		41	0.23 (− 0.01 to 0.47)	0.087	15.65%
*Length*		Subgroup analysis: *F*_1, 11.04_ = 0.00,* p* = .965			
Short (1–3 month)		71	0.21 (0.00–0.43)*	0.098	51.88%
Long (4–6 month)		43	0.22 (0.03–0.40)*	0.072	21.02%

Note: *CI* confidence interval, *95% CI exclude zero, *HIIT* high-intensity interval training

**Fig. 3:**
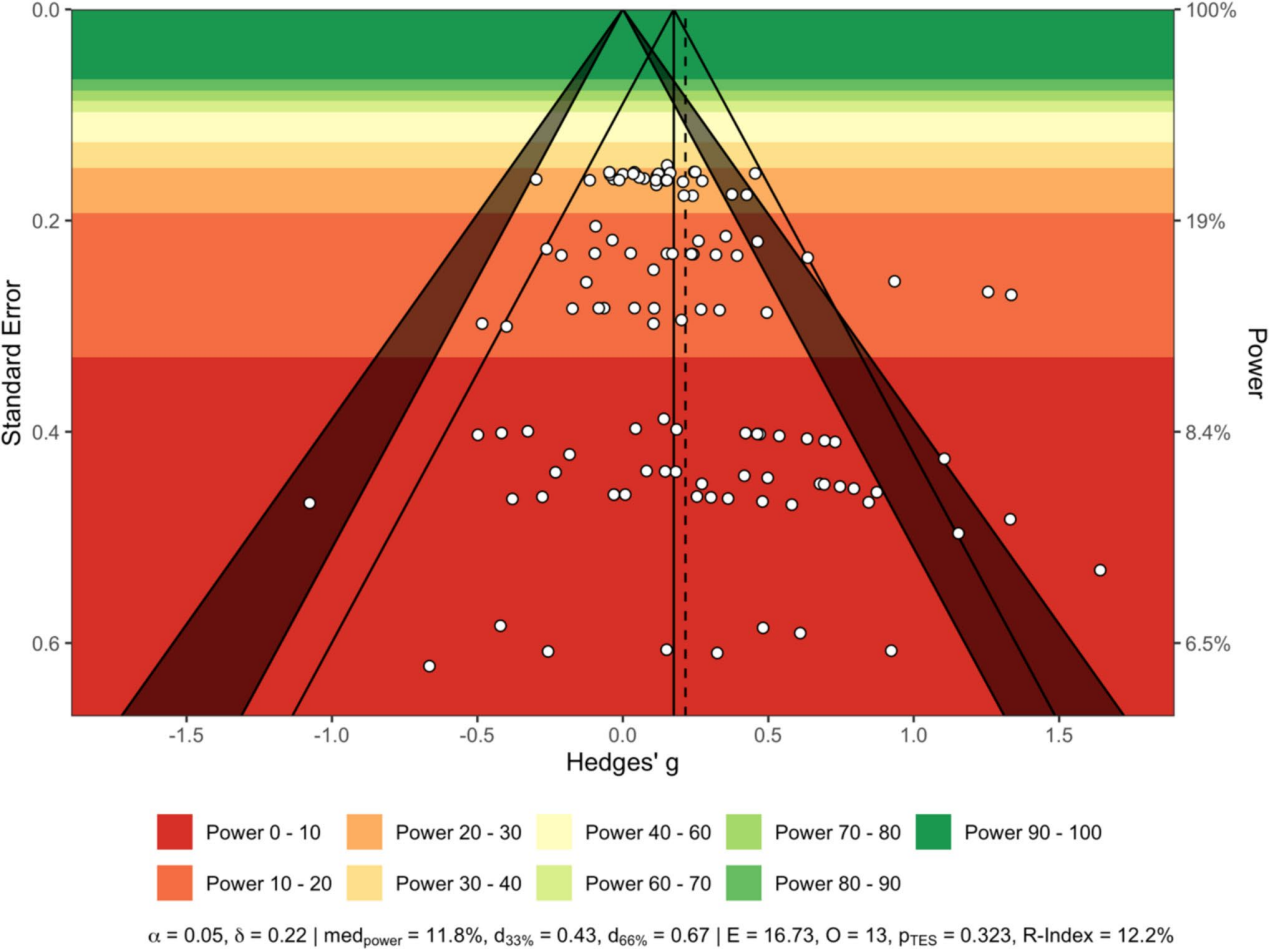
Sunset funnel plot

The power-enhanced sunset funnel plot (Fig. 3) revealed an asymmetrical distribution of studies. Smaller studies with larger standard errors tended to report larger effects compared to more precise studies with smaller standard errors, indicating potential small-study effects. Assuming a true effect size of δ = 0.22 (α = 0.05), the median power of the included studies was 11.8%. Thirteen nominally significant effect sizes were observed, while 16.73 significant effect sizes were expected (*p*_TES_ = 0.32), suggesting no evidence of excess significance. The expected replicability of findings is low (R-index = 12.2%). To achieve median power levels of 33% and 66%, effect sizes of 0.43 and 0.67 would be required, respectively. Egger’s regression test indicated no publication bias existed for all included studies (*p* = 0.27).

Sensitivity analysis revealed two outliers in residuals [40, 48]. Following the removal of these two effect sizes, the pooled effect size showed a slight further reduction (*g* = 0.21, 95% CI [0.09, 0.34], *p* = 0.002). Nine effect sizes exceeded three times the mean Cook’s distance [13, 39–41, 47, 51, 54, 55]. Following their removal, the pooled effect size experienced a slight further reduction (*g* = 0.17, 95% CI [0.08, 0.25], *p* = 0.001).

### Subgroup analysis

The results of subgroup analyses are outlined in Table 3. The corresponding forest plots and funnel plots are presented in Supplement Figs. 1–8 and Supplement Figs. 9–16, respectively.

### Cognitive function domains

Subgroup analysis of cognitive function domains was not significant (*F* = 1.34, *p* = *0.5*49). Exercise significantly enhanced self-report cognitive function (*g* = 0.29, 95% CI [0.10, 0.48]), basic cognitive function (*g* = 0.23, 95% CI [0.06, 0.41]) and high-order cognitive function (*g* = 0.12, 95% CI [0.00, 0.23]). However, no significant effect of exercise was observed on memory (*g* = 0.09, 95% CI [ – 0.16, 0.34]).

### Sample characteristic

Subgroup analysis of age was not significant (*F* = 2.00, *p* = *0.3*69). Exercise had positive effects for cognitive function in breast cancer patients and survivors aged below 60 years (*g* = 0.23, 95% CI [0.07, 0.39]). However, no significant effect of exercise on cognitive function was observed in breast cancer patients and survivors aged over 60 years (*g* = 0.11, 95% CI [ – 0.33, 0.56]).

Additionally, subgroup analysis of timing of exercise was not significant (*F* = 0.70, *p* = *0.5*85). Exercise had positive effects for cognitive function in breast cancer survivors after treatment (*g* = 0.21, 95% CI [0.07, 0.35]). However, no significant effect of exercise on cognitive function was observed in breast cancer patients during treatment (*g* = 0.25, 95% CI [ – 0.05, 0.56]) and mix (*g* = 0.03, 95% CI [ – 1.92, 1.98]).

### Exercise prescription variables

Subgroup analysis of exercise frequency was not significant (*F* = 2.25, *p* = 0.171). Exercise with moderate frequency (3–4 times per week) (*g* = 0.27, 95% CI [0.11, 0.43]) significantly enhanced cognitive function. However, no significant effect of exercise on cognitive function was observed with high (> 4 times per week) (*g* = 0.40, 95% CI [ – 0.50, 1.30]) and low frequency (1–2 times per week) (*g* = 0.08, 95% CI [ – 0.06, 0.23]).

Additionally, subgroup analysis of exercise intensity was not significant (*F*_3, 2.39_ = 1.59, *p* = 0.385). Exercise at vigorous intensity significantly enhanced cognitive function (*g* = 0.20, 95% CI [0.02, 0.38]) and not reported (*g* = 0.35, 95% CI [0.01, 0.70]). However, moderate (*g* = 0.10, 95% CI [ – 0.24, 0.43]) and light intensity (*g* =  – 0.07, 95% CI [ – 1.40, 1.27]) did not demonstrate significant effects.

Regarding the exercise type, subgroup analysis of exercise type was not significant (*F* = 15.70, *p* = 0.053). HIIT (*g* = 0.46, 95% CI [0.06, 0.85]) and multi-component exercise (*g* = 0.30, 95% CI [0.09, 0.51]) conferred significant benefits for cognitive function in breast cancer patients. However, aerobic exercise (*g* = 0.09, 95% CI [ – 0.12, 0.29]) and resistance exercise (*g* = 0.00, 95% CI [ – 0.22, 0.21]) did not demonstrate significant effect.

Furthermore, subgroup analysis of exercise intervention session time was not significant (*F* = 1.03, *p* = 0.407). Exercise with short (≤ 30 min) (*g* = 0.43, 95% CI [ – 0.38, 1.25]), moderate (31–60 min) (*g* = 0.12, 95% CI [ – 0.02, 0.26]) and long session time (> 60 min) (*g* = 0.23, 95% CI [ – 0.01, 0.47]) did not demonstrate significant effect.

Finally, subgroup analysis of the length of exercise intervention was not significant (*F* = 0.00, *p* = 0.965). Both short-term (1–3 months) (*g* = 0.21, 95% CI [0.00, 0.43]) and long-term (4–6 months) (*g* = 0.22, 95% CI [0.03, 0.40]) exercise interventions yielded positive benefits for cognitive function.

## Discussion

To our knowledge, this is the first meta-analysis specifically examining the effects of exercise on cognitive function in both breast cancer patients and survivors as well as analyzing cognitive function domains, sample characteristics, and exercise regimens as moderators specifically within the breast cancer population. The results demonstrated that exercise had a positive, statistically significant, small effect size on cognitive function in populations diagnosed with breast cancer. This beneficial effect remained consistent across all examined moderators.

### Overall effect

Our results indicate that exercise effectively improves cognitive function in breast cancer patients and survivors, aligning with meta-analyses on various populations including healthy older adults [56], individuals with chronic diseases [57], Alzheimer’s patients [58], and prostate cancer patients [59]. Although the exact mechanisms by which exercise improves cognitive function in breast cancer patients remain unclear, brain-derived neurotrophic factor (BDNF) has been considered a potential mediator. BDNF, as one of the most prevalent neurotrophic factors in the brain, plays a crucial role in neuronal growth, development, and synaptic plasticity regulation [60], with increased BDNF levels associated with improved cognitive function [61]. Exercise has been shown to elevate BDNF levels in breast cancer survivors [62].

Beyond BDNF, multiple mechanisms likely contributed to the cognitive benefits of exercise. CRCI is thought to be associated with increased inflammatory markers and negative psychological states, reflecting its multifactorial nature and complexity. Research indicates that exercise may reduce pro-inflammatory cytokines that have been demonstrated to be closely associated with cognitive impairment [63]. Additionally, exercise effectively mitigates negative emotional states such as depression, anxiety, and stress, which frequently impair cognitive performance [64]. This suggests that exercise interventions may ameliorate these negative impacts across multiple pathways, thereby effectively enhancing cognitive function in breast cancer patients and survivors.

### Cognitive function domains

Although the subgroup analysis revealed no moderation effect, further analysis yielded a significant positive effect with small to moderate effect sizes for self-report cognitive function (*g* = 0.32), basic cognitive functions (*g* = 0.24) and high-order cognitive functions (*g* = 0.11). Self-report cognitive function is important for breast cancer patients [65, 66], as it reflects subtle deficits in cognitive function [67], impacting their daily lives [65], as well as emotional and fatigue-related symptoms [66]. Additionally, basic cognitive functions were the most affected domains in these CRCI symptoms [68]. Our results suggest that exercise may effectively mitigate these cognitive deficits, offering a promising approach to alleviate the long-term negative cognitive effects associated with chemotherapy in breast cancer patients and survivors.

Exercise had a positive impact on high-order cognitive function in breast cancer patients and survivors, which is important as it is highly associated with daily activities [69]. Breast cancer patients undergoing chemotherapy often experience impairments in high-order cognitive function, with prevalence as high as 86.6% among survivors [70, 71]. Our study found exercise can effectively improve high-order cognitive function, suggesting implications for future interventions. However, benefits were limited to memory, inconsistent with prior reports in adults with mild cognitive impairment [72]. This discrepancy warrants further investigation in future studies.

### Sample characteristics

Although the subgroup analysis revealed no moderation effect for age, our results revealed a significant small to moderate effect size for individuals under 60 years of age, but not for individuals over 60 years. This finding is consistent with previous research on cognitive function in other clinical populations with depression [73], but not with findings in healthy populations [74] and the observed inconsistencies may contribute to variability in the participants’ results. Notably, most of the effect sizes were conducted among individuals under 60 years old (*n* = 96), compared to only 14 effect sizes for those aged over 60 years. This highlights a gap in our understanding of the effects of exercise on breast cancer patients and survivors aged 60 and older.

A novel feature of this study is the examination of breast cancer patients at different timings of exercise. Subgroup analysis revealed that the timing of exercise did not moderate the exercise outcomes. However, a significant small to moderate benefit from exercise for breast cancer survivors was observed, aligning with previous meta-analyses [25]. For breast cancer patients undergoing treatment, exercise showed limited impact on cognitive function. This discrepancy may be attributed to excessive heterogeneity [75], as this study exhibited the highest level of heterogeneity among all subgroups (*I*^2^ = 71.00%). Consequently, further research is recommended to elucidate the cognitive benefits of exercise for breast cancer patients at different timings of exercise.

### Exercise regimens

Although the subgroup analysis revealed no moderation effect for exercise frequency, further analysis yielded a significant positive effect with a small to moderate effect size for moderate frequency (3–4 times per week), but not low (< 3 times per week) and high frequency (> 4 time per week). These results are in line with patients with major depressive disorder [76], but contrary to older adults who observed that any frequency of exercise positively impacts cognitive function, with high frequency exercise yielding the greatest cognitive benefits [56]. This result may be attributed to differences in the populations investigated. Our review has highlighted the potential role of exercise frequency, facilitating further empirical exploration of the dose–response relationship to investigate the impact of exercise frequency on cognitive function in cancer patients or survivors.

Regarding exercise intensity, the subgroup analysis revealed no moderation effect of exercise intensity on cognitive function, while further analysis demonstrated a significant positive effect with a small to moderate effect size for vigorous intensity, but not light and moderate intensity. These findings partially align with previous research on healthy older adults [18, 56] and patients with depression [73], which found that moderate and vigorous exercise has benefits on cognitive function. However, our results did not indicate significant benefits of moderate intensity exercise for breast cancer patients and survivors. Notably, intensity categorized as “not reported” exhibited significant small to moderate effects, contributing a large number of effect sizes (*n* = 42) in our study. Many included studies implemented yoga or qigong exercises [41, 42, 48], generally considered light or moderate intensity. However, the absence of heart rate data or specific exercise prescriptions in these studies makes it challenging to accurately assess intensity levels. Future research, particularly those employing these exercise types, could consider monitoring heart rate or utilizing ratings of perceived exertion to comprehensively elucidate the impact of exercise intensity on cognitive function.

Similarly, the subgroup analysis revealed no moderation effect of exercise type on cognitive function, while our results revealed a significant effect with a moderate to large effect size for HIIT, as well as a moderate effect size for multi-component exercise. Recent systematic reviews have demonstrated positive effects of HIIT on executive function [77], and our research is among the first to identify HIIT as a beneficial exercise type in this context. Additionally, our findings for multi-component exercise align with previous studies on cognitive function [56] and executive function [18] in older adults. This exercise type may be particularly appropriate for the cancer population, as it has been a prescribed regimen especially pertinent for older adults [78].

Our study found no moderation effect of exercise session time. We observed no significant effects across various session time levels, which aligns with previous research on the quality of life of cancer patients [79]. This suggests that different exercise session times may yield comparable benefits for cognitive function, thereby limiting the efficacy of session time as a potential moderating factor. Finally, both exercise lengths (i.e., 1–3 months or 4–6 months) showed similar effects in breast cancer patients, which is consistent with previous research on non-cancer populations [18, 80], suggesting a range of 1–6 months is recommended.

### Strengths and limitations

The major strength of this study lies in its utilization of multilevel meta-analysis to address the hierarchical structure of data and heterogeneity. Additionally, by exclusively targeting studies with randomized controlled trials, the results can be interpreted more in terms of causal relationships. Finally, by encompassing both breast cancer patients and survivors, along with conducting various moderation analyses, our study offers more specific exercise recommendations. However, several limitations warrant consideration. Our study solely examines the isolated effect of exercise, thereby overlooking potentially effective strategies that synergistically combine exercise with other interventions such as dietary supplementation or cognitive training. Second, the limited number of effect sizes in some moderation levels undermines the robustness of the evidence and necessitates cautious interpretation of the results. Third, due to limited information in some of the prior research reports, we were unable to differentiate the timing of exercise interventions within the treatment stage, which might affect the results. Fourth, our study did not specifically investigate cancer stage or severity due to limited information provided in the research. Fourth, our study did not specifically investigate cancer stage or severity due to limited information provided in the research. Finally, the utilization of only three databases (PubMed, Scopus, and Web of Science) during our literature collection process may affect the comprehensiveness of our research. Although these databases contain substantial relevant research literature, incorporating additional databases such as CINAHL, Embase, or PsycINFO could potentially facilitate the discovery of other pertinent literature. These limitations highlight the need for future empirical studies to explore combined intervention approaches, examine the effects of exercise interventions on breast cancer patients at different cancer stages, and carefully examine the timing of exercise interventions within the treatment stage to better understand their effects on breast cancer patients at different treatment phases.

## Conclusion

Our findings highlight the beneficial effects of exercise on cognitive function in breast cancer patients and survivors. The analysis revealed no moderation effects across various exercise parameters, suggesting that diverse exercise regimens may be effective for this population. Furthermore, the observed cognitive benefits appear to be independent of age. Despite these encouraging results, research in this area remains limited, underscoring the need for further investigation into the effects of exercise on cognitive function in this population.

## Supplementary Information

Below is the link to the electronic supplementary material.

Supplementary file1 (DOCX 125711 KB)

## Data Availability

All data generated or analyzed during this three-level meta-analysis are included in the article and supplementary information.

## Declarations

### Statement on the welfare of animals

This article does not contain any studies with human participants or animals performed by any of the authors.
