# Supplementary material for: Effect of exercise interventions on cognitive function in breast cancer patients and survivors: a systematic review with meta-analysis
Source: Breast Cancer. 2025 Aug 18;32(6):1180–96. doi: 10.1007/s12282-025-01757-9 (PMC12552352; doi:10.1007/s12282-025-01757-9)
Supplement: Supplementary file 1 — Supplementary file1 (DOCX 125711 KB) [file 12282_2025_1757_MOESM1_ESM.docx]

**Effect of Exercise Interventions on Cognitive Function in Breast Cancer Patients and Survivors: A Systematic Review with Meta-Analysis**

Chen-Sin Hung, Yi-Ting Cheng, Ruei-Hong Li, Charles H. Hillman,

Neha P. Gothe, Feng-Tzu Chen, Hsing-Jung Yeh, Fei-Fei Ren, Yu-Kai Chang

**Supplemental Content**

[Supplemental Methods	5](#_Toc176198715)

[Data Coding	5](#_Toc176198716)

[Data Analysis	5](#_Toc176198717)

[Supplement Table 1 Search Based upon Three Primary Terms (Exercise, Cognitive Function, Breast Cancer)	8](#_Toc176198718)

[(1) Search terms in PubMed, Last search date: 25 December 2024	7](#_Toc176198719)

[(2) Search terms in Scopus, Last search date: 25 December 2024	8](#_Toc176198720)

[(3) Search terms in Web of Science, Last search date: 25 December 2024	9](#_Toc176198721)

[Supplement Table 2	10](#_Toc176198722)

[(1)	Physiotherapy Evidence Database Scale Criteria	10](#_Toc176198723)

[(2)	Grading of Recommendations Assessment, Development and Evaluation Criteria	11](#_Toc176198724)

[Supplement Table 3 Grading of Recommendations Assessment, Development and Evaluation assessment	12](#_Toc176198725)

[(1) Cognitive function domain	12](#_Toc176198726)

[(2) Age	13](#_Toc176198727)

[(3) Chemotherapy stage	14](#_Toc176198728)

[(4) Exercise frequency	15](#_Toc176198729)

[(5) Exercise intensity	16](#_Toc176198730)

[(6) Exercise type	17](#_Toc176198731)

[(7) Exercise session time	18](#_Toc176198732)

[(8) Exercise length	19](#_Toc176198733)

[Supplement Figure 1 The forest plot of cognitive function domain	20](#_Toc176198734)

[(1) Self-report cognitive function	20](#_Toc176198735)

[(2) Basic cognitive function	21](#_Toc176198736)

[(3) High-order cognitive function	22](#_Toc176198737)

[(4) Memory	23](#_Toc176198738)

[Supplement Figure 2 The forest plot of age	24](#_Toc176198739)

[(1) Middle-aged (≤ 60 yrs)	24](#_Toc176198740)

[(2) Older adults (> 60 yrs)	25](#_Toc176198741)

[Supplement Figure 3 The forest plot of chemotherapy stage	26](#_Toc176198742)

[(1) Patients (during treatment)	26](#_Toc176198743)

[(2) Survivors (after treatment)	27](#_Toc176198744)

[(3) Mixed (patients and survivors)	28](#_Toc176198745)

[Supplement Figure 4 The forest plot of exercise frequency	29](#_Toc176198746)

[(1) Low exercise frequency (1-2 times)	29](#_Toc176198747)

[(2) Moderate exercise frequency (3-4 times)	30](#_Toc176198748)

[(3) High exercise frequency (> 4 times)	31](#_Toc176198749)

[Supplement Figure 5 The forest plot of exercise intensity	32](#_Toc176198750)

[(1) Light exercise intensity	32](#_Toc176198751)

[(2) Moderate exercise intensity	33](#_Toc176198752)

[(3) Vigorous exercise intensity	34](#_Toc176198753)

[(4) Not reported exercise intensity	35](#_Toc176198754)

[Supplement Figure 6 The forest plot of exercise type	36](#_Toc176198755)

[(1) Aerobic exercise	36](#_Toc176198756)

[(2) Resistance exercise	37](#_Toc176198757)

[(3) HIIT	38](#_Toc176198758)

[(4) Multi-component exercise	39](#_Toc176198759)

[Supplement Figure 7 The forest plot of exercise session time	40](#_Toc176198760)

[(1) Short exercise session time (≤ 30 min)	40](#_Toc176198761)

[(2) Moderate exercise session time (31-60 min)	41](#_Toc176198762)

[(3) Long exercise session time (> 60 min)	42](#_Toc176198763)

[Supplement Figure 8 The forest plot of exercise length	43](#_Toc176198764)

[(1) Short exercise length (1-3 month)	43](#_Toc176198765)

[(2) Long exercise length (4-6 month)	44](#_Toc176198766)

[Supplement Figure 9 The funnel plot of cognitive function domain	45](#_Toc176198767)

[(1) Self-report cognitive function	45](#_Toc176198768)

[(2) Basic cognitive function	46](#_Toc176198769)

[(3) High-order cognitive function	47](#_Toc176198770)

[(4) Memory	48](#_Toc176198771)

[Supplement Figure 10 The funnel plot of age	49](#_Toc176198772)

[(1) Middle-aged (≤ 60 yrs)	49](#_Toc176198773)

[(2) Older adults (> 60 yrs)	50](#_Toc176198774)

[Supplement Figure 11 The funnel plot of chemotherapy stage	51](#_Toc176198775)

[(1) Patients (during treatment)	51](#_Toc176198776)

[(2) Survivors (after treatment)	52](#_Toc176198777)

[(3) Mixed (patients and survivors)	53](#_Toc176198778)

[Supplement Figure 12 The funnel plot of exercise frequency	54](#_Toc176198779)

[(1) Low exercise frequency (1-2 times)	54](#_Toc176198780)

[(2) Moderate exercise frequency (3-4 times)	55](#_Toc176198781)

[(3) High exercise frequency (< 4 times)	56](#_Toc176198782)

[Supplement Figure 13 The funnel plot of exercise intensity	57](#_Toc176198783)

[(1) Light exercise intensity	57](#_Toc176198784)

[(2) Moderate exercise intensity	58](#_Toc176198785)

[(3) Vigorous exercise intensity	59](#_Toc176198786)

[(4) Not reported exercise intensity	60](#_Toc176198787)

[Supplement Figure 14 The funnel plot of exercise type	61](#_Toc176198788)

[(1) Aerobic exercise	61](#_Toc176198789)

[(2) Resistance exercise	62](#_Toc176198790)

[(3) HIIT	63](#_Toc176198791)

[(4) Multi-component exercise	64](#_Toc176198792)

[Supplement Figure 15 The funnel plot of exercise session time	65](#_Toc176198793)

[(1) Short exercise session time (≤ 30 min)	65](#_Toc176198794)

[(2) Moderate exercise session time (31-60 min)	66](#_Toc176198795)

[(3) Long exercise session time (< 60 min)	67](#_Toc176198796)

[Supplement Figure 16	68](#_Toc176198797)

[(1) Short exercise length (1-3 month)	68](#_Toc176198798)

[(2) Long exercise length (4-6 month)	69](#_Toc176198799)

# Supplemental Methods

## Data Coding

In the domain of cognitive function, coding was conducted according to self-report cognitive function, basic cognitive function, high-order cognitive function, and memory. Self-report cognitive function refers to subjective cognitive function measured through questionnaires. Basic cognitive function includes attention, processing speed, and motor functioning (Meffert & Dahme, 1993), whereas higher-order cognitive function encompasses executive function (Diamond, 2013) and learning (Levine, 2009).

Regarding sample characteristics, age and chemotherapy stage were coded. Age was coded as either middle-aged (46-60 years) or older adults (> 60 years). Additionally, the chemotherapy stage was coded based on treatment completion as patients (during treatment), survivors (after treatment), or mixed (included patients and survivors). Regarding exercise characteristics, components of frequency, intensity, type, session time, and length were included. Exercise frequency was coded as low (≤ 2 times/week), moderate (3-4 times/week), or high (5-7 times/week) (Northey et al., 2018). Exercise intensity was coded as light (2-2.9 METs; ≤ 39% HRR/VO2max; ≤ 63% HRmax, ≤ 49% 1-RM, RPE ≤ 11 on the Borg scale), moderate (3-5.9 METs; 40-59% HRR/VO2max; 64-76% HRmax, 50-69% 1-RM, RPE value 12-13 on the Borg scale), vigorous (≥ 6 METs; ≥ 60% HRR/VO2max; ≥ 77% HRmax; ≥ 70% 1-RM, RPE value ≥ 14 on the Borg scale), or not report intensity (American College of Sports Medicine, 2022). Exercise type was coded based on the following types: aerobic exercise, resistance exercise, high-intensity interval training (HIIT), and multi-component exercise. Session time was coded as short (≤ 30 minutes), moderate (31-60 minutes), or long (> 60 minutes) session (Baillet et al., 2010), and exercise length was coded as short-term (1-3 months) or long-term (4-6 months) length (Colcombe & Kramer, 2003). Notably, if the exercise dosage gradually increases, it will be coded based on the exercise characteristics of the final target dosage, while if the exercise intensity remains within a specific range, the median value will be used for coding.

## Data Analysis

Data Synthesis and Analysis

We conducted a multi-level meta-analysis using the “metafor” package (version 4.1.2) (Viechtbauer, 2010) in R (R Core Team, 2013). This advanced modeling approach was selected to address the dependency of effect sizes, as multiple effect sizes were nested within individual studies. This multi-level meta-analysis framework accounts for three levels of variance: sampling variance (level 1), within-study variance (level 2), and between-study variance (level 3) (Cheung, 2014).

Effect sizes were calculated using the standard mean difference (i.e., Hedges’ g) for between-groups pretest-posttest designs [30], with the assumption of a moderate correlation between pretest and post-test measures (r = .5) (Skvarc & Fuller-Tyszkiewicz, 2024). We assessed heterogeneity using the I2 statistic and Cochrane’s Q-test. Significance in heterogeneity was considered if the p-value of the Q-test was less than 0.1. I2 values were categorized as follows: ≤ 25% (low), 26%-50% (mild), 51%-75% (moderate), and > 75% (high) heterogeneity (Migliavaca et al., 2022). Publication bias was evaluated through visual inspection of a power-enhanced sunset funnel plot. A multilevel variant of Egger’s regression test was conducted to assess the presence of the small-study effect. Sensitivity analyses were conducted to identify and quantify the influence of outliers and influential meta-analytic reviews (i.e., studentized residuals and Cook’s distance) on the pooled effect estimates (Viechtbauer & Cheung, 2010).

To address potential bias from studies reporting multiple outcomes, we implemented robust variance estimation (RVE) using the “clubSandwich” method. This approach prevents studies with numerous outcome measures from exerting disproportionate influence on the overall effect size estimation by: (1) correcting for dependence between effect sizes within studies, (2) generating cluster-robust variance estimates, (3) employing small-sample corrections for improved inference, and (4) effectively balancing each study's contribution regardless of how many outcomes they report (Pustejovsky & Tipton, 2022).

Subgroup analyses were executed to investigate moderators of the observed effects, including cognitive function domains (self-report cognitive function, basic cognitive function, high-order cognitive function, and memory), age (middle-aged and older adults), timing of exercise (patients, survivors, and mixed), exercise frequency (low, moderate, and high), intensity (low, moderate, vigorous, and not reported), type (aerobic exercise, resistance exercise, high-intensity interval training [HIIT], and multi-component exercise), session time (short, moderate, and long), length (short and long).

# Supplement Table 1 Search Based upon Three Primary Terms (Exercise, Cognitive Function, Breast Cancer)

## (1) Search terms in PubMed, Last search date: 25 December 2024 (7918)

| No. | Query | Limitation/Filters |
| --- | --- | --- |
| 1 | exercise* OR “physical activity” OR “physical exercise” OR aerobic OR running OR walking OR jogging OR cycling OR “anaerobic exercise” OR resistance OR “strength exercise” OR “weight exercise” OR sport* OR qigong OR “martial art” OR “mind-body exercise” OR Taiji OR “Tai chi” OR Taichi OR baduanjin OR yoga | TITLE/  ABSTRACT |
| 2 | cognition* OR cognitive OR “cognitive function*” OR “cognitive performance” OR “global cognition” OR “central executive” OR “executive process*” OR “executive control*” OR “cognitive control*” OR “executive function” OR inhibition OR “inhibitory control” OR “interference control” OR “working memory” OR updating OR “cognitive flexibility” OR shifting OR switching OR planning OR “problem solving” OR problem-solving OR reasoning OR attention OR concentration OR vigilance OR “processing speed*” OR “cognitive speed” OR memory OR “decision making” OR intelligence OR language OR learning OR psychomotor OR neurocogniti* | TITLE/  ABSTRACT |
| 3 | “breast cancer” OR “mammary cancer” OR “breast tumor” OR “mammary tumor” | TITLE/  ABSTRACT |
| 4 | 1 AND 2 AND 3 |  |

## (2) Search terms in Scopus, Last search date: 25 December 2024 (21399)

| No. | Query | Limitation/Filters |
| --- | --- | --- |
| 1 | exercise* OR “physical activity” OR “physical fitness” OR “physical exercise” OR aerobic OR running OR walking OR jogging OR cycling OR “anaerobic exercise” OR resistance OR “strength exercise” OR “weight exercise” OR sports OR sport* OR qigong OR “martial art” OR “mind-body exercise” OR taiji OR “tai chi” OR taichi OR baduanjin OR yoga | TITLE/  ABSTRACT/  KEYWORD |
| 2 | cognition* OR cognitive OR “cognitive function*” OR “cognitive performance” OR “global cognition” OR “central executive” OR “executive process*” OR “executive control*” OR “cognitive control*” OR “executive function” OR inhibition OR “inhibitory control” OR “interference control” OR “working memory” OR updating OR “cognitive flexibility” OR shifting OR switching OR planning OR “problem solving” OR problem-solving OR reasoning OR attention OR concentration OR vigilance OR “processing speed*” OR “cognitive speed” OR memory OR “decision making” OR intelligence OR language OR learning OR psychomotor OR neurocogniti* | TITLE/  ABSTRACT/  KEYWORD |
| 3 | “breast cancer” OR “mammary cancer” OR “malignant breast tumor” | TITLE/  ABSTRACT/  KEYWORD |
| 4 | 1 AND 2 AND 3 |  |

## (3) Search terms in Web of Science, Last search date: 25 December 2024 (32268)

| No. | Query | Limitation/Filters |
| --- | --- | --- |
| 1 | exercise* OR “physical activity” OR “physical fitness” OR “physical exercise” OR aerobic OR running OR walking OR jogging OR cycling OR “anaerobic exercise” OR resistance OR “strength exercise” OR “weight exercise” OR sports OR sport* OR qigong OR “martial art” OR “mind-body exercise” OR Taiji OR “Tai chi” OR Taichi OR baduanjin OR yoga | TOPIC |
| 2 | cognition* OR cognitive OR “cognitive function*” OR “cognitive performance” OR “global cognition” OR “central executive” OR “executive process*” OR “executive control*” OR “cognitive control*” OR “executive function” OR inhibition OR “inhibitory control” OR “interference control” OR “working memory” OR updating OR “cognitive flexibility” OR shifting OR switching OR planning OR “problem solving” OR problem-solving OR reasoning OR attention OR concentration OR vigilance OR “processing speed*” OR “cognitive speed” OR memory OR “decision making” OR intelligence OR language OR learning OR psychomotor OR neurocogniti* | TOPIC |
| 3 | “breast cancer” OR “mammary cancer” OR “malignant breast tumor” | TOPIC |
| 4 | 1 AND 2 AND 3 |  |

# Supplement Table 2

(1) **Physiotherapy Evidence Database Scale Criteria**

| 1 | Explicit participant inclusion criteria |
| --- | --- |
| 2 | Randomization of participants within each group |
| 3 | Allocation concealment |
| 4 | Baseline similarity in cognitive function between groups |
| 5 | Blinding of participants |
| 6 | Blinding of treatment providers |
| 7 | Blinding of outcome assessors |
| 8 | Cognitive function measurements for at least 85% of participants |
| 9 | Intention-to-treat analysis |
| 10 | Reporting of at least one between-group comparison of cognitive function results |
| 11 | Provision of at least one point measure and variability measure for cognitive function |

## (2) Grading of Recommendations Assessment, Development and Evaluation Criteria

| Criteria | Detail |
| --- | --- |
| Risk of bias | - Not serious: low risk of bias - Serious: moderate risk of bias - Very serious: high risk of bias |
| Inconsistency | - Not serious: *I²* lower than 50% - Serious: *I²* between 50% and 75% - Very serious: *I²* higher than 75% |
| Indirectness | - Not serious: The absence of indirectness in the PICO principle or methodological aspects - Serious: The presence of indirectness in any one of component of the PICO principle - Very serious: The presence of indirectness in more than one component of the PICO principle |
| Imprecision | - Not serious: (a) Meet the optimal information size (OIS = 1570) and (b) the overall 95% confidence interval is less than 0.5 - Serious: If either (a) or (b) criteria was violated - Very serious: Both criteria were violated |
| Other consideration | - Not serious: Egger regression test was not significant - Serious: Egger regression test was significant |

# Supplement Table 3 Grading of Recommendations Assessment, Development and Evaluation assessment

## (1) Cognitive function domain

| **Certainty assessment** | | | | | | | **№ of patients** | | **Effect** | | **Certainty** |
| --- | --- | --- | --- | --- | --- | --- | --- | --- | --- | --- | --- |
| **№ of studies** | **Study design** | **Risk of bias** | **Inconsistency** | **Indirectness** | **Imprecision** | **Other considerations** | **Exercise** | **Control** | **Relative (95% CI)** | **Absolute (95% CI** |  |
| Self-report cognitive function | | | | | | | | | | | |
| 18 | randomised trials | serious^a^ | serious^b^ | not serious | not serious | none | 1408 | 1360 | SMD **0.29 SD higher** (0.10 higher to 0.48 higher) | | ⨁⨁◯◯ Low |
| Basic cognitive function | | | | | | | | | | | |
| 6 | randomised trials | serious^a^ | not serious | not serious | serious^c^ | none | 502 | 512 | SMD **0.24 SD higher** (0.07 higher to 0.4 higher) | | ⨁⨁◯◯ Low |
| High-order cognitive function | | | | | | | | | | | |
| 10 | randomised trials | not serious | not serious | not serious | not serious | none | 1258 | 1246 | SMD **0.12 SD higher** (0 to 0.23 higher) | | ⨁⨁⨁⨁ High |
| Memory | | | | | | | | | | | |
| 6 | randomised trials | serious^a^ | not serious | not serious | not serious | publication bias strongly suspected^d^ | 897 | 877 | SMD **0.09 SD higher** (0.16 lower to 0.34 higher) | | ⨁⨁◯◯ Low |

**CI:** confidence interval; **SMD:** standardised mean difference

a. not serious: low risk of bias, serious: moderate risk of bias, very serious: high risk of bias

b. not serious: I² lower than 50%, serious: I² between 50% and 75%, very serious: I² higher than 75%

c. not serious: (a) Meet the optimal information size (OIS = 1570) and (b) the overall 95% confidence interval was less than 0.5, serious: If either (a) or (b) criteria was violated, very serious: Both criteria were violated

d. Egger regression test was significant

## (2) Age

| **Certainty assessment** | | | | | | | **№ of patients** | | **Effect** | | **Certainty** |
| --- | --- | --- | --- | --- | --- | --- | --- | --- | --- | --- | --- |
| **№ of studies** | **Study design** | **Risk of bias** | **Inconsistency** | **Indirectness** | **Imprecision** | **Other considerations** | **Exercise** | **Control** | **Relative (95% CI)** | **Absolute (95% CI** |  |
| Middle-aged (≤ 60 yrs) | | | | | | | | | | | |
| 19 | randomised trials | serious^a^ | serious^b^ | not serious | not serious | none | 3559 | 3491 | SMD **0.23 SD higher** (0.07 higher to 0.39 higher) | | ⨁⨁◯◯ Low |
| Older adults (> 60 yrs) | | | | | | | | | | | |
| 2 | randomised trials | not serious | not serious | not serious | very serious^c^ | none | 506 | 504 | SMD **0.11 SD higher** (0.33 lower to 0.56 higher) | | ⨁⨁◯◯ Low |

**CI:** confidence interval; **SMD:** standardised mean difference

a. not serious: low risk of bias, serious: moderate risk of bias, very serious: high risk of bias

b. not serious: I² lower than 50%, serious: I² between 50% and 75%, very serious: I² higher than 75%

c. not serious: (a) Meet the optimal information size (OIS = 1570) and (b) the overall 95% confidence interval was less than 0.5, serious: If either (a) or (b) criteria was violated, very serious: Both criteria were violated

d. Egger regression test was significant

## (3) Timing of exercise

| **Certainty assessment** | | | | | | | **№ of patients** | | **Effect** | | **Certainty** |
| --- | --- | --- | --- | --- | --- | --- | --- | --- | --- | --- | --- |
| **№ of studies** | **Study design** | **Risk of bias** | **Inconsistency** | **Indirectness** | **Imprecision** | **Other considerations** | **Exercise** | **Control** | **Relative (95% CI)** | **Absolute (95% CI)** |  |
| Patients (during treatment) | | | | | | | | | | | |
| 10 | randomised trials | not serious | serious^b^ | not serious | serious^c^ | none | 1163 | 1117 | SMD **0.25 SD higher** (0.05 lower to 0.56 higher) | | ⨁⨁◯◯ Low |
| Survivors (after treatment) | | | | | | | | | | | |
| 9 | randomised trials | serious^a^ | not serious | not serious | not serious | none | 2430 | 2409 | SMD **0.24 SD higher** (0.1 higher to 0.38 higher) | | ⨁⨁⨁◯ Moderate |
| Mixed (patients and survivors) | | | | | | | | | | | |
| 2 | randomised trials | not serious | not serious | not serious | very serious^c^ | none | 472 | 469 | SMD **0.03 SD higher** (-1.92 higher to 1.98 higher) | | ⨁⨁◯◯ Low |

**CI:** confidence interval; **SMD:** standardised mean difference

a. not serious: low risk of bias, serious: moderate risk of bias, very serious: high risk of bias

b. not serious: I² lower than 50%, serious: I² between 50% and 75%, very serious: I² higher than 75%

c. not serious: (a) Meet the optimal information size (OIS = 1570) and (b) the overall 95% confidence interval was less than 0.5, serious: If either (a) or (b) criteria was violated, very serious: Both criteria were violated

d. Egger regression test was significant

## (4) Exercise frequency

| **Certainty assessment** | | | | | | | **№ of patients** | | **Effect** | | **Certainty** |
| --- | --- | --- | --- | --- | --- | --- | --- | --- | --- | --- | --- |
| **№ of studies** | **Study design** | **Risk of bias** | **Inconsistency** | **Indirectness** | **Imprecision** | **Other considerations** | **Exercise** | **Control** | **Relative (95% CI)** | **Absolute (95% CI)** |  |
| Low (1-2 times) | | | | | | | | | | | |
| 9 | randomised trials | not serious | not serious | not serious | not serious | none | 2345 | 2329 | SMD **0.08 SD higher** (0.06 lower to 0.23 higher) | | ⨁⨁⨁⨁ High |
| Moderate (3-4 times) | | | | | | | | | | | |
| 8 | randomised trials | not serious | not serious | not serious | not serious | none | 1069 | 1045 | SMD **0.27 SD higher** (0.11 higher to 0.43 higher) | | ⨁⨁⨁⨁ High |
| High (> 4 times) | | | | | | | | | | | |
| 4 | randomised trials | serious^a^ | serious^b^ | not serious | very serious^c^ | none | 651 | 621 | SMD **0.40 SD higher** (0.50 lower to 1.3 higher) | | ⨁◯◯◯ Very low |

**CI:** confidence interval; **SMD:** standardised mean difference

a. not serious: low risk of bias, serious: moderate risk of bias, very serious: high risk of bias

b. not serious: I² lower than 50%, serious: I² between 50% and 75%, very serious: I² higher than 75%

c. not serious: (a) Meet the optimal information size (OIS = 1570) and (b) the overall 95% confidence interval was less than 0.5, serious: If either (a) or (b) criteria was violated, very serious: Both criteria were violated

d. Egger regression test was significant

## (5) Exercise intensity

| **Certainty assessment** | | | | | | | **№ of patients** | | **Effect** | | **Certainty** |
| --- | --- | --- | --- | --- | --- | --- | --- | --- | --- | --- | --- |
| **№ of studies** | **Study design** | **Risk of bias** | **Inconsistency** | **Indirectness** | **Imprecision** | **Other considerations** | **Exercise** | **Control** | **Relative (95% CI)** | **Absolute (95% CI)** |  |
| Light | | | | | | | | | | | |
| 2 | randomised trials | not serious | not serious | not serious | very serious^c^ | none | 136 | 155 | SMD **0.07 SD lower** (1.40 lower to 1.27 higher) | | ⨁⨁◯◯ Low |
| Moderate | | | | | | | | | | | |
| 4 | randomised trials | not serious | not serious | not serious | very serious^c^ | none | 764 | 780 | SMD **0.10 SD higher** (0.24 lower to 0.43 higher) | | ⨁⨁◯◯ Low |
| Vigorous | | | | | | | | | | | |
| 7 | randomised trials | not serious | not serious | not serious | not serious | none | 2211 | 2144 | SMD **0.20 SD higher** (0.02 higher to 0.38 higher) | | ⨁⨁⨁⨁ High |
| Not reported | | | | | | | | | | | |
| 9 | randomised trials | serious^a^ | serious^b^ | not serious | serious^c^ | none | 954 | 916 | SMD **0.35 SD higher** (0.01 higher to 0.70 higher) | | ⨁◯◯◯ Very low |

**CI:** confidence interval; **SMD:** standardised mean difference

a. not serious: low risk of bias, serious: moderate risk of bias, very serious: high risk of bias

b. not serious: I² lower than 50%, serious: I² between 50% and 75%, very serious: I² higher than 75%

c. not serious: (a) Meet the optimal information size (OIS = 1570) and (b) the overall 95% confidence interval was less than 0.5, serious: If either (a) or (b) criteria was violated, very serious: Both criteria were violated

d. Egger regression test was significant

## (6) Exercise type

| **Certainty assessment** | | | | | | | **№ of patients** | | | **Effect** | | **Certainty** |
| --- | --- | --- | --- | --- | --- | --- | --- | --- | --- | --- | --- | --- |
| **№ of studies** | **Study design** | **Risk of bias** | **Inconsistency** | **Indirectness** | **Imprecision** | **Other considerations** | **Exercise** | **Control** | | **Relative (95% CI)** | **Absolute (95% CI)** |  |
| Aerobic exercise | | | | | | | | | | | | |
| 5 | randomised trials | not serious | not serious | not serious | not serious | none | 842 | 840 | | SMD **0.09 SD higher** (0.12 lower to 0.29 higher) | | ⨁⨁⨁⨁ High |
| Resistance exercise | | | | | | | | | | | | |
| 3 | randomised trials | not serious | not serious | not serious | serious^c^ | publication bias strongly suspected^d^ | 401 | | 385 | SMD **0 SD**  (0.22 lower to 0.21 higher) | | ⨁⨁◯◯ Low |
| HIIT | | | | | | | | | | | | |
| 1 | randomised trials | not serious | not serious | not serious | very serious^c^ | none | 24 | | 24 | SMD **0.46 SD higher** (0.06 higher to 0.85 higher) | | ⨁⨁◯◯ Low |
| Multi-component exercise | | | | | | | | | | | | |
| 14 | randomised trials | serious^a^ | serious^b^ | not serious | not serious | none | 2798 | | 2746 | SMD **0.35 SD higher** (0.12 higher to 0.58 higher) | | ⨁⨁◯◯ Low |

**CI:** confidence interval; **SMD:** standardised mean difference

a. not serious: low risk of bias, serious: moderate risk of bias, very serious: high risk of bias

b. not serious: I² lower than 50%, serious: I² between 50% and 75%, very serious: I² higher than 75%

c. not serious: (a) Meet the optimal information size (OIS = 1570) and (b) the overall 95% confidence interval was less than 0.5, serious: If either (a) or (b) criteria was violated, very serious: Both criteria were violated

d. Egger regression test was significant

## (7) Exercise session time

| **Certainty assessment** | | | | | | | **№ of patients** | | **Effect** | | **Certainty** |
| --- | --- | --- | --- | --- | --- | --- | --- | --- | --- | --- | --- |
| **№ of studies** | **Study design** | **Risk of bias** | **Inconsistency** | **Indirectness** | **Imprecision** | **Other considerations** | **Exercise** | **Control** | **Relative (95% CI)** | **Absolute (95% CI)** |  |
| Short (≤ 30 min) | | | | | | | | | | | |
| 4 | randomised trials | serious^a^ | serious^b^ | not serious | very serious^c^ | none | 665 | 639 | SMD **0.43 SD higher** (0.38 lower to 1.25 higher) | | ⨁◯◯◯ Very low |
| Moderate (31-60 min) | | | | | | | | | | | |
| 11 | randomised trials | not serious | not serious | not serious | not serious | none | 1533 | 1481 | SMD **0.12 SD higher** (0.02 lower to 0.26 higher) | | ⨁⨁⨁⨁ High |
| Long (> 60 min) | | | | | | | | | | | |
| 6 | randomised trials | not serious | not serious | not serious | not serious | none | 1867 | 1875 | SMD **0.23 SD higher** (0.01 lower to 0.47 higher) | | ⨁⨁⨁⨁ High |

**CI:** confidence interval; **SMD:** standardised mean difference

a. not serious: low risk of bias, serious: moderate risk of bias, very serious: high risk of bias

b. not serious: I² lower than 50%, serious: I² between 50% and 75%, very serious: I² higher than 75%

c. not serious: (a) Meet the optimal information size (OIS = 1570) and (b) the overall 95% confidence interval was less than 0.5, serious: If either (a) or (b) criteria was violated, very serious: Both criteria were violated

d. Egger regression test was significant

## (8) Exercise length

| **Certainty assessment** | | | | | | | **№ of patients** | | **Effect** | | **Certainty** |
| --- | --- | --- | --- | --- | --- | --- | --- | --- | --- | --- | --- |
| **№ of studies** | **Study design** | **Risk of bias** | **Inconsistency** | **Indirectness** | **Imprecision** | **Other considerations** | **Exercise** | **Control** | **Relative (95% CI)** | **Absolute (95% CI)** |  |
| Short (1-3 month) | | | | | | | | | | | |
| 14 | randomised trials | serious^a^ | serious^b^ | not serious | not serious | none | 1599 | 1558 | SMD **0.21 SD higher** (0.00 higher to 0.43 higher) | | ⨁⨁◯◯ Low |
| Long (4-6 month) | | | | | | | | | | | |
| 7 | randomised trials | not serious | not serious | not serious | not serious | none | 2340 | 2293 | SMD **0.22 SD higher** (0.03 higher to 0.40 higher) | | ⨁⨁⨁⨁ High |

**CI:** confidence interval; **SMD:** standardised mean difference

a. not serious: low risk of bias, serious: moderate risk of bias, very serious: high risk of bias

b. not serious: I² lower than 50%, serious: I² between 50% and 75%, very serious: I² higher than 75%

c. not serious: (a) Meet the optimal information size (OIS = 1570) and (b) the overall 95% confidence interval was less than 0.5, serious: If either (a) or (b) criteria was violated, very serious: Both criteria were violated

d. Egger regression test was significant

# Supplement Figure 1 The forest plot of cognitive function domain

## (1) Self-report cognitive function

## (2) Basic cognitive function

## (3) High-order cognitive function

## (4) Memory

# Supplement Figure 2 The forest plot of age

## (1) Middle-aged (≤ 60 yrs)

## (2) Older adults (> 60 yrs)

# Supplement Figure 3 The forest plot of timing of exercise

## (1) Patients (during treatment)

## (2) Survivors (after treatment)

## (3) Mixed (patients and survivors)

# Supplement Figure 4 The forest plot of exercise frequency

## (1) Low exercise frequency (1-2 times)

## (2) Moderate exercise frequency (3-4 times)

## (3) High exercise frequency (> 4 times)

# Supplement Figure 5 The forest plot of exercise intensity

## (1) Light exercise intensity

## (2) Moderate exercise intensity

## (3) Vigorous exercise intensity

## (4) Not reported exercise intensity

# Supplement Figure 6 The forest plot of exercise type

## (1) Aerobic exercise

## (2) Resistance exercise

## (3) HIIT

## (4) Multi-component exercise

# Supplement Figure 7 The forest plot of exercise session time

## (1) Short exercise session time (≤ 30 min)

## (2) Moderate exercise session time (31-60 min)

## (3) Long exercise session time (> 60 min)

# Supplement Figure 8 The forest plot of exercise length

## (1) Short exercise length (1-3 month)

## (2) Long exercise length (4-6 month)

# Supplement Figure 9 The funnel plot of cognitive function domain

## (1) Self-report cognitive function

## (2) Basic cognitive function

## (3) High-order cognitive function

## (4) Memory

# Supplement Figure 10 The funnel plot of age

## (1) Middle-aged (≤ 60 yrs)

## (2) Older adults (> 60 yrs)

# Supplement Figure 11 The funnel plot of timing of exercise

## (1) Patients (during treatment)

## (2) Survivors (after treatment)

## (3) Mixed (patients and survivors)

# Supplement Figure 12 The funnel plot of exercise frequency

## (1) Low exercise frequency (1-2 times)

## (2) Moderate exercise frequency (3-4 times)

## (3) High exercise frequency (< 4 times)

# Supplement Figure 13 The funnel plot of exercise intensity

## (1) Light exercise intensity

## (2) Moderate exercise intensity

## (3) Vigorous exercise intensity

## (4) Not reported exercise intensity

# Supplement Figure 14 The funnel plot of exercise type

## (1) Aerobic exercise

## (2) Resistance exercise

## (3) HIIT

## (4) Multi-component exercise

# Supplement Figure 15 The funnel plot of exercise session time

## (1) Short exercise session time (≤ 30 min)

## (2) Moderate exercise session time (31-60 min)

## (3) Long exercise session time (< 60 min)

# Supplement Figure 16 The funnel plot of exercise length

## (1) Short exercise length (1-3 month)

## (2) Long exercise length (4-6 month)

**Reference**

American College of Sports Medicine. (2022). *ACSM's guidelines for exercise testing and prescription* (G. Liguori, Ed. 11th ed.). Wolters Kluwer. <https://shop.lww.com/ACSM-s-Guidelines-for-Exercise-Testing-and-Prescription/p/9781975150181>

Baillet, A., Zeboulon, N., Gossec, L., Combescure, C., Bodin, L. A., Juvin, R., Dougados, M., & Gaudin, P. (2010). Efficacy of cardiorespiratory aerobic exercise in rheumatoid arthritis: meta‐analysis of randomized controlled trials. *Arthritis Care Res 62*(7), 984–992.

Cheung, M. W. L. (2014). Modeling dependent effect sizes with three-level meta-analyses: a structural equation modeling approach. *Psychol Methods*, *19*(2), 211.

Colcombe, S., & Kramer, A. F. (2003). Fitness effects on the cognitive function of older adults: a meta-analytic study. *Psychol Sci*, *14*(2), 125–130.

Diamond, A. (2013). Executive functions. *Annu Rev Psychol*, *64*, 135–168. <https://doi.org/10.1146/annurev-psych-113011-143750>

Levine, M. D. (2009). Differences in learning and neurodevelopmental function in school-age children. In *Developmental-behavioral pediatrics* (pp. 535–546). Elsevier.

Meffert, H.-J., & Dahme, B. (1993). Impairment in basic cognitive functioning: attention, concentration, mental flexibility. *Cerebral Damage Before and After Cardiac Surgery*, 185–193.

Migliavaca, C. B., Stein, C., Colpani, V., Barker, T. H., Ziegelmann, P. K., Munn, Z., Falavigna, M., & Group, P. E. R. S. R. M. (2022). Meta‐analysis of prevalence: I 2 statistic and how to deal with heterogeneity. *Research synthesis methods*, *13*(3), 363–367.

Northey, J. M., Cherbuin, N., Pumpa, K. L., Smee, D. J., & Rattray, B. (2018). Exercise interventions for cognitive function in adults older than 50: a systematic review with meta-analysis. *Br J Sports Med*, *52*(3), 154–160.

Pustejovsky, J. E., & Tipton, E. (2022). Meta-analysis with Robust Variance Estimation: Expanding the Range of Working Models. *Prevention Science*, *23*(3), 425–438. <https://doi.org/10.1007/s11121-021-01246-3>

R Core Team, R. (2013). R: a language and environment for statistical computing.

Skvarc, D. R., & Fuller-Tyszkiewicz, M. (2024). Calculating repeated-measures meta-analytic effects for continuous outcomes: a tutorial on pretest–posttest-controlled designs. *Adv Methods Pract Psychol Sci*, *7*(1), 25152459231217238.

Viechtbauer, W. (2010). Conducting meta-analyses in R with the metafor package. *J Stat Softw*, *36*(3), 1 – 48. <https://doi.org/10.18637/jss.v036.i03>

Viechtbauer, W., & Cheung, M. W. L. (2010). Outlier and influence diagnostics for meta‐analysis. *Res Synth Methods*, *1*(2), 112–125.
